# Comprehensive evaluation of genes related to basement membrane in hepatocellular carcinoma

**DOI:** 10.18632/aging.205923

**Published:** 2024-06-12

**Authors:** Guojing Wu, Fei Li, Danyan Guo, Kaiwen Xi, Dayong Zheng, Ruichao Huang, Xiuqiong Wu, Aimin Li, Xinhui Liu

**Affiliations:** 1 Southern Medical University Hospital of Integrated Traditional Chinese and Western Medicine, Southern Medical University, Guangzhou 510315, China; 2 Cancer Center, Southern Medical University, Guangzhou 510315, China; 3 Heshan Hospital of Traditional Chinese Medicine, Jiangmen 529000, China

**Keywords:** basement membrane-related genes, hepatocellular carcinoma, prognostic model, tumor microenvironment, quantitative real-time PCR

## Abstract

In all mammals, the basement membrane serves as a pivotal extracellular matrix. Hepatocellular carcinoma (HCC) is a challenge among numerous cancer types shaped by basement membrane-related genes (BMGs). Our research established an innovative prognostic model that is highly accurate in its prediction of HCC prognoses and immunotherapy efficacy to summarize the crucial role of BMGs in HCC. We obtained HCC transcriptome analysis data and corresponding clinical data from The Cancer Genome Atlas (TCGA). To augment our dataset, we incorporated 222 differentially expressed BMGs identified from relevant literature. A weighted gene coexpression network analysis (WGCNA) of 10158 genes demonstrated four modules that were connected to HCC. Additionally, 66 genes that are found at the intersection of BMGs and HCC-related genes were designated as hub HCC-related BMGs. MMP1, ITGA2, P3H1, and CTSA comprise the novel model that was engineered using univariate and multivariate Cox regression analysis. Furthermore, the International Cancer Genome Consortium (ICGC) and Gene Expression Omnibus (GEO) datasets encouraged the BMs model’s validity. The overall survival (OS) of individuals with HCC may be precisely predicted in the TCGA and ICGC databases utilizing the BMs model. A nomogram based on the model was created in the TCGA database at similar time, and displayed a favorable discriminating ability for HCC. Particularly, when compared to the patients at an elevated risk, the patients with a low-risk profile presented different tumor microenvironment (TME) and hallmark pathways. Moreover, we discovered that a lower risk score of HCC patients would display a greater response to immunotherapy. Finally, quantitative real-time PCR (qRT-PCR) experiments were used to verify the expression patterns of BMs model. In summary, BMs model demonstrated efficacy in prognosticating the survival probability of HCC patients and their immunotherapeutic responsiveness.

## INTRODUCTION

Liver cancer ranks as the sixth most common cancer globally and stands third in cancer-related mortality, as reported by the World Health Organization [1]. Among primary liver malignancies, hepatocellular carcinoma (HCC) stands as the prevailing histological subtype, accounting for approximately 70-85% of all instances [2]. Despite the fact that HCC is a major health concern, there are very few effective interventions available, and surgical resection remains the best treatment for HCC [3]. Over the past ten years, there have been only modest advancements in systemic therapies for liver cancer [4]. As a result, comprehending the biological mechanisms that drive HCC malignancy becomes imperative in order to devise more potent therapeutic approaches.

Basement membranes (BMs), an ancient extracellular matrix that appeared with the emergence of multicellular animals [5], are predominately composed of collagen, laminin, and non-collagenous glycoproteins [6]. Nidogen and heparan sulfate proteoglycans act as network-bridging proteins between type IV collagen molecules and laminin [7], and assist in the building of BMs along tissues by providing scaffolding structure [8]. Moreover, the BMs also incorporate “matricellular proteins” that serve specialized roles tailored to specific tissues, though they do not participate in the constitution or structural cohesion [9]. Functions of BMs including blood filtration, muscle homeostasis, controlling angiogenesis and tumor growth, mechanical stress resistant and storing growth factors and cytokines [7]. Studies have shown that germline variants in approximately 30 basement membrane-related genes (BMGs) can lead to genetic disorders [10]. Furthermore, emerging evidence suggests that disruptions in the synthesis and degradation of BMs proteins may be associated with fibrosis, diabetes, and cancer [11–13]. Additionally, collagen XIX, a lesser-known member of the FACIT family, has demonstrated the ability to suppress angiogenesis, thus inhibiting the new blood vessel formation and potentially impeding tumor cell invasion [14]. Additionally, the interaction between integrin α3β1 and laminins is crucial to tumor cell proliferation initiation and maintenance [15]. Studies have indicated that CD8^+^ T cells infiltrate tumor lesions by secreting granzyme B, which promotes the remodeling of the BMs of tumor blood vessels, facilitating their migration [16] that means BMs may be related to the immune microenvironment in HCC. However, BMs are not well understood in HCC in terms of their role and status. Thus, it is quite crucial to explicate whether BMGs are effective at predicting HCC prognosis and immunotherapy response for HCC patients.

In the present study, an extensive investigation of characteristics and biomarkers associated with BMGs was carried out, as they will provide a means to explore HCC immune infiltration and monitor the response to immunotherapy. First, we constructed coexpression networks of tumor samples and identified the intersecting genes of BMGs by weighted gene coexpression network analysis (WGCNA). Then a model based on BMGs was established and its prognostic value was assessed in predicting HCC patients’ outcome and response to immunotherapy and chemotherapy. In conclusion, our research provides novel insights that could lead to improved prognosis and treatment outcomes for HCC patients in the coming years.

## RESULTS

### Identification and enrichment analysis of catheter holder modules associated with HCC

**Figure 1:**
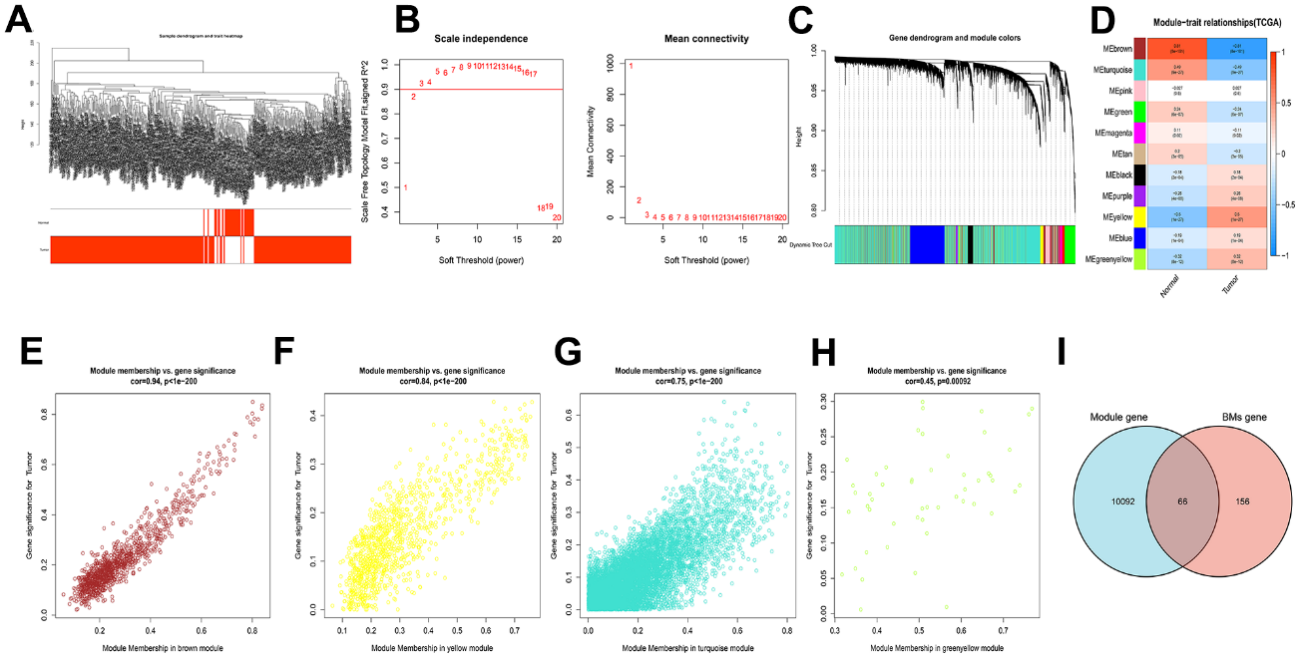
**WGCNA analysis results of 10158 genes of HCC.** (**A**) Clustering dendrogram of HCC samples. (**B**) The scale–free fit index for soft–thresholding powers. (**C**) A dendrogram of the differentially expressed genes clustering based on different metrics. (**D**) A heatmap showing the correlation between the gene module and associated traits. (**E**–**H**) Scatter plots of module eigengenes in brown, yellow, turquoise and greenyellow modules. (**I**) Venn diagram showed the intersection of genes of HCC and BMGs.

In the TCGA-LIHC cohort, the brown module (R^2^ = -0.81, P = 8e−101), yellow module (R^2^ = 0.5, P = 1e−27), turquoise module (R^2^ = -0.49, P = 9e−27) and greenyellow module (R^2^ = 0.32, P = 8e−12) were extremely correlated with tumor samples among the 11 modules (Figure 1A–1H). These four modules were identified as hub modules. Subsequently, there are 10158 genes that related to tumor were identified from the emergence of the above modules. Then, there are 66 common genes between hub module genes and BMGs for further study (Figure 1I).

In this part, 66 pivotal genes that could determine HCC progression and metastasis by BMs were explored for future research.

### GO and KEGG enrichment analysis of BMGs in HCC

**Figure 2:**
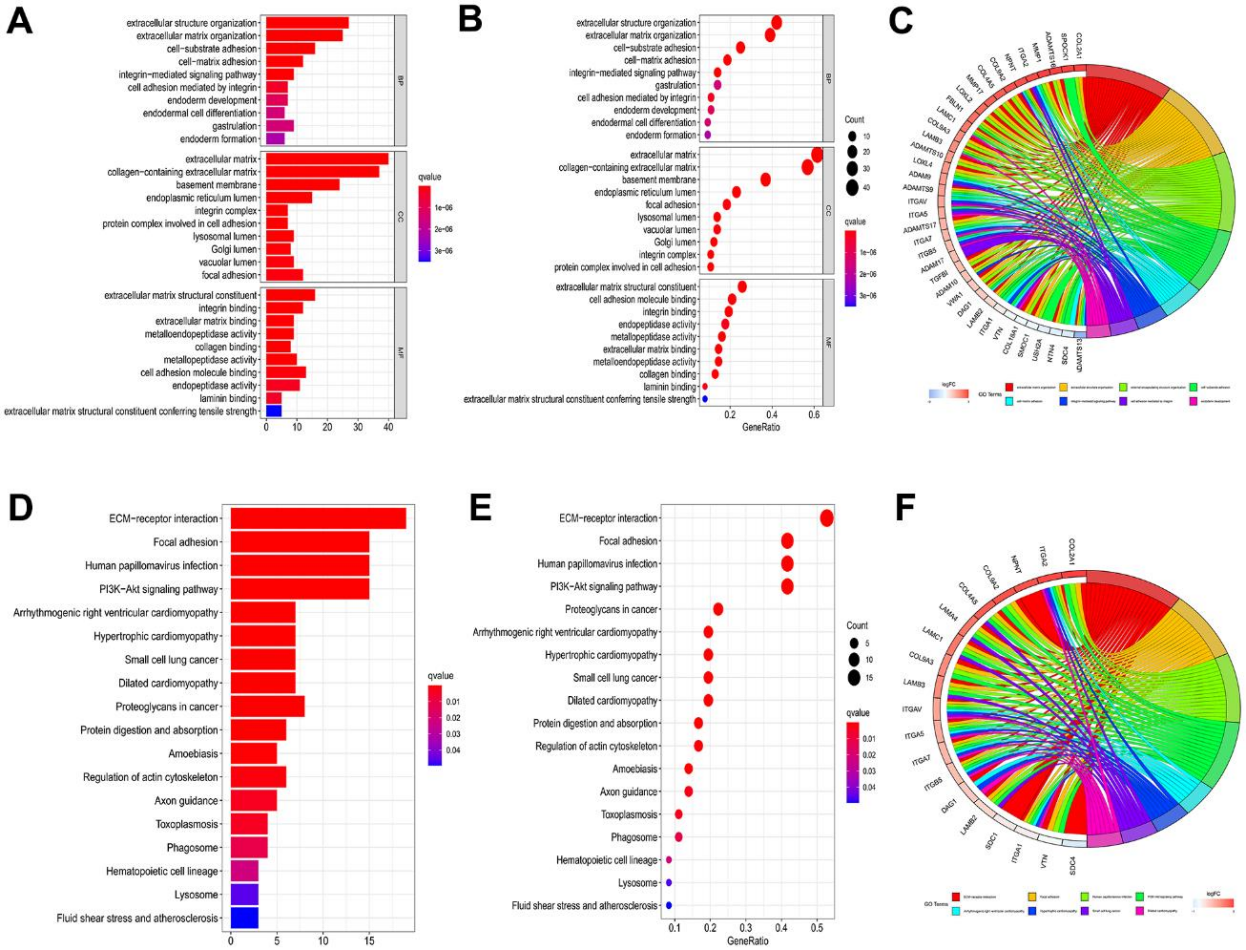
**GO and KEGG enrichment analysis to 66 genes of intersection.** Barplot (**A**), bubble plot (**B**) and chord diagram (**C**) of GO analyses of genes of intersection. Barplot (**D**), bubble plot (**E**) and chord diagram (**F**) of KEGG analyses of genes of intersection.

The “clusterProfiler” R package was employed for GO enrichment analysis to elucidate the relevant biological processes and molecular structures, and the correlated pathways were acquired applying KEGG enrichment analysis. As illustrated by GO enrichment analysis, the intersection of genes was closely related to the extracellular matrix and collagen-containing extracellular matrix in the Cellular Component (CC). The enriched Biological Processes (BP) primarily encompassed extracellular structure organization and extracellular matrix organization. In terms of Molecular Function (MF), changes were observed in extracellular structural constituent and cell adhesion molecule binding (Figure 2A–2C). KEGG enrichment analysis revealed significant gene intersections associated with extracellular matrix (ECM)-receptor interaction, Focal adhesion, Human papillomavirus infection, and PI3K-AKT signaling pathway (Figure 2D–2F).

Taken together, this part offered new ideas and clew to construct future exploration from CC, BP and MF by utilizing the GO enrichment analysis and the KEGG enrichment analysis.

### Multi-omics landscape of BMs in HCC

**Figure 3:**
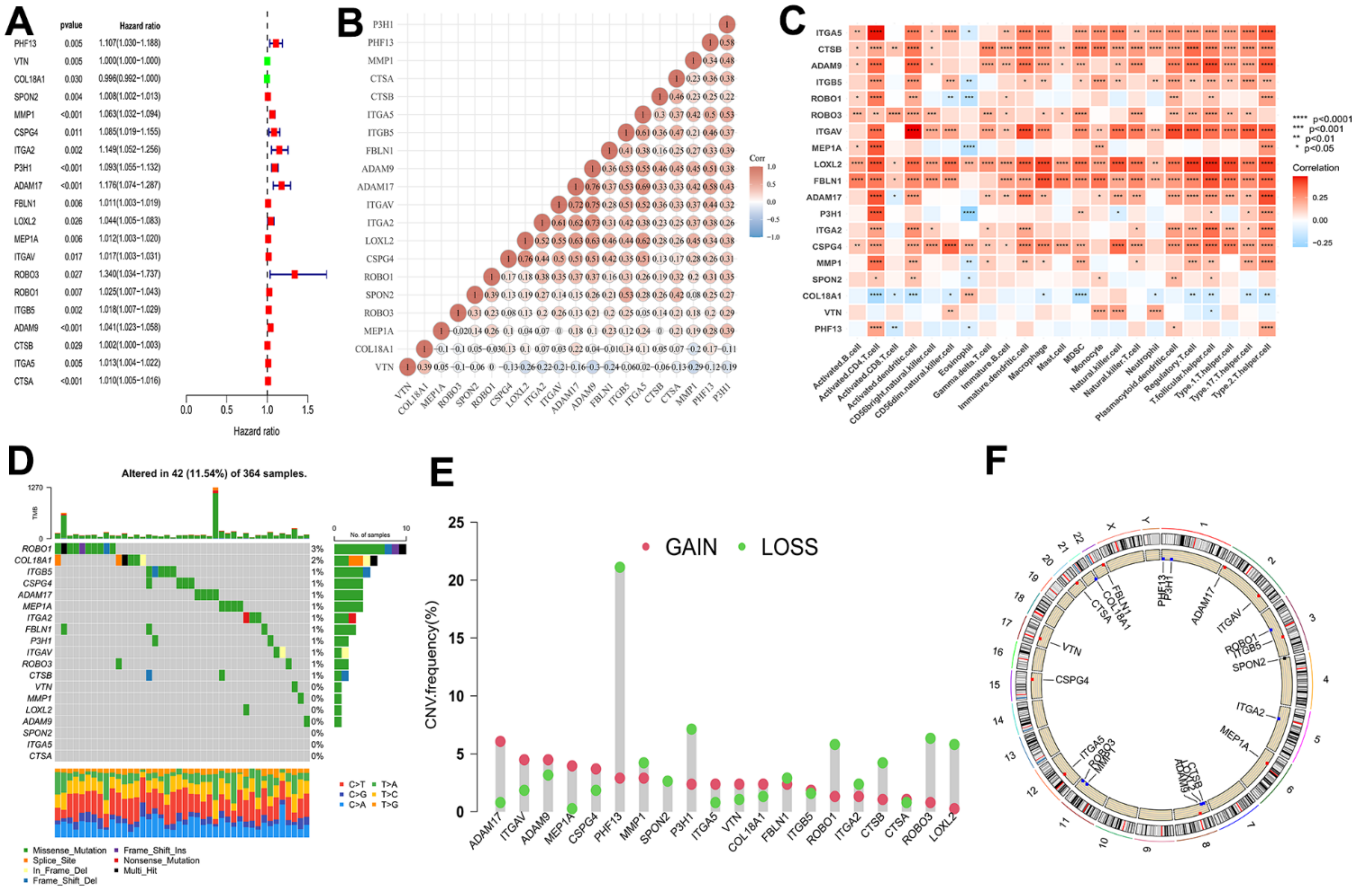
**Multi-omics landscape by prognostic genes.** (**A**) Univariate Cox regression analysis of genes. (**B**) Significant positive correlation between genes related to prognostic. (**C**) The correlation between genes related to prognostic and the infiltration level of 23 immune cells. (**D**) The mutation frequency of 20 BMGs in TCGA-LIHC cohort. Each column of the figure represents an individual patient. (**E**) The CNV variation frequency of BMGs (red and green plots separately represent CNV gain and CNV loss). (**F**) Locations of CNV alterations in BMGs on 23 chromosomes. * p < 0.05; ** p < 0.01; *** p < 0.001; **** p < 0.0001.

We have shown Cox regression analysis of all 66 common genes (Supplementary Table 1) and identified 20 BMGs that related to prognostic by univariate Cox regression analysis (Figure 3A). Next, in TME of HCC, the relationship between BMGs and immune cell infiltration was investigated. As illustrated in Figure 3B, a significant positive correlation observed among the BMGs. Moreover, notable positive associations were observed between BMGs and the infiltration of immune cells in HCC individuals (Figure 3C). CNV is a DNA fragment in the human genome with a copy size of 1kB to 1MB. It is associated with tumorigenesis and tumor progression, including activation of oncogenes, inactivation of oncogenes, genomic heterogeneity and molecular phenotype [17, 18]. We analyzed BMGs for gene amplification and deletion frequencies. We displayed a waterfall plot illustrating a relatively low mutation rate of the 20 genes in the TCGA cohort (Figure 3D). ADAM17, ITGAV, ADAM9 and MEP1A had widespread CNV amplification, while PHF13, P3H1, ROBO3, LOXL2 presented prevalent CNV deletions (Figure 3E), and the locations of CNV alterations on chromosomes showed as Figure 3F.

In a word, 20 genes correlated with BMs were screened from 66 intersection of genes, which revealed a noteworthy and statistically significant positive correlation with immune cell infiltration and involved in ADAM17, ITGAV, ADAM9 and MEP1A that conducted widespread amplification and PHF13, P3H1, ROBO3, LOXL2 that implemented prevalent CNV deletions.

### BMs model development and validation in HCC

**Figure 4:**
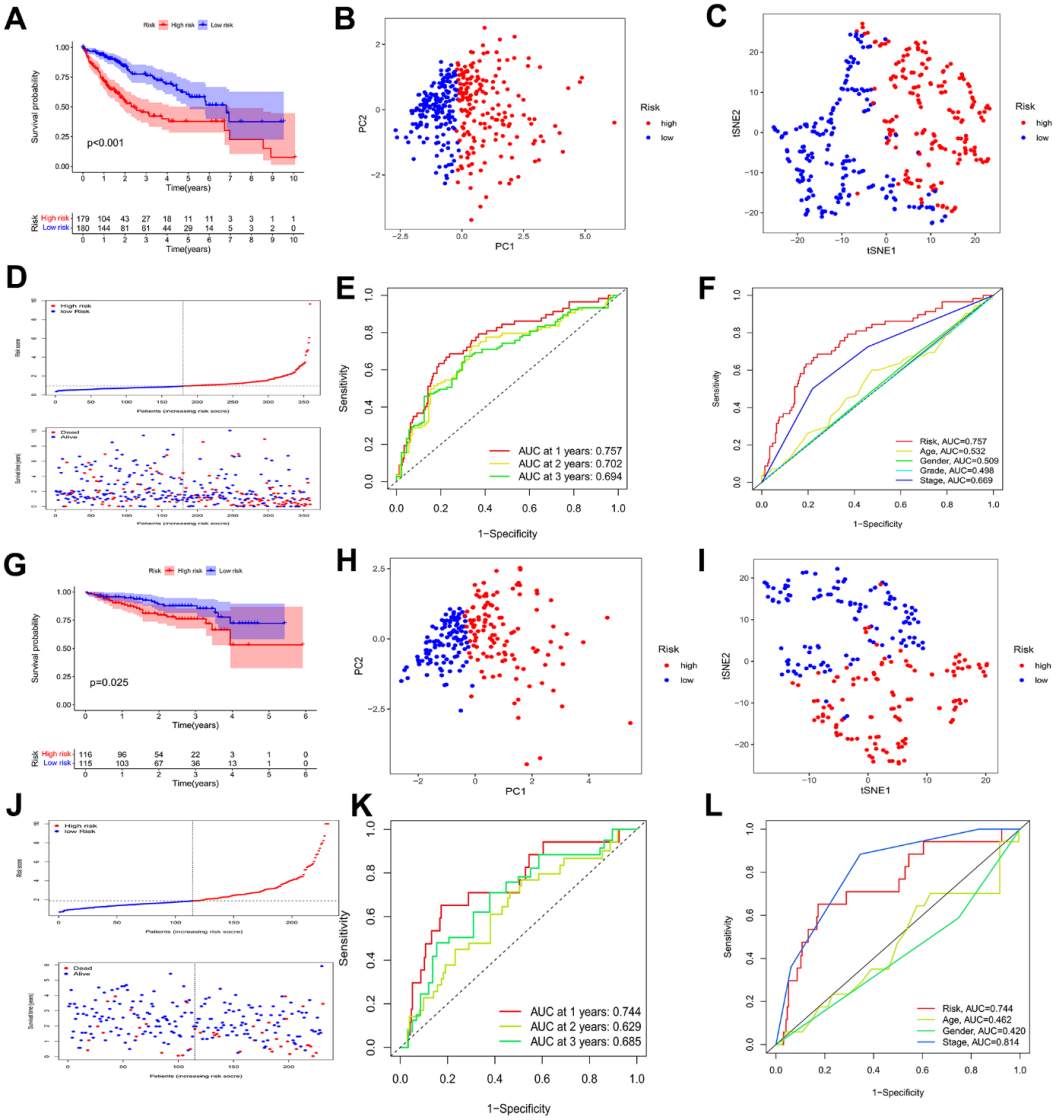
**Identification of a prognostic risk model for HCC patients.** (**A**) Kaplan–Meier curve of HCC patients in the TCGA cohort. (**B**, **C**) PCA and t-SNE analysis showing a remarkable difference in transcriptomes between the two risk categories in the TCGA cohort. (**D**) Scatter plots showing the risk score distribution and patient survival status in TCGA cohort. (**E**) ROC curves to predict the sensitivity and specificity of 1-, 2-, 3-year survival according to the risk score in the TCGA cohort. (**F**) Clinical ROC analysis in the TCGA cohort. (**G**) Kaplan–Meier curve of HCC patients in the ICGC cohort. (**H**, **I**) PCA and t-SNE analysis showing a remarkable difference in transcriptomes between the two risk categories in the ICGC cohort. (**J**) Scatter plots showing the risk score distribution and patient survival status in the ICGC cohort. (**K**) ROC curves to predict the sensitivity and specificity of 1-, 2-, 3-year survival according the risk score in the ICGC cohort. (**L**) Clinical ROC analysis in the ICGC cohort.

We applied multivariate Cox regression analysis to identify 4 genes (MMP1, ITGA2, P3H1, CTSA) to establish a risk model. The excellent formula, Risk score = (0.238*MMP1) + (0.212*ITGA2) + (0.400*P3H1) + (0.240*CTSA), was utilized to evaluate the risk score. The midpoint risk score serves for a criterion that can categorized into high- and low-risk profiles. The ICGC queue serves as an external validation set. Both the TCGA cohort (Figure 4A) and the ICGC cohort (Figure 4G) demonstrated that patients with lower risk scores experienced more favorable outcomes, as depicted by the Kaplan-Meier curve. The four core risk genes MMP1, ITGA2, P3H1, and CTSA were utilized for PCA and t-SNE analysis which indicate that the BMs model shows bright differentiation (Figure 4B, 4C, 4H, 4I). The patients at an elevated risk were characterized by higher mortality and decreased survival from risk curves. The number of patients in the high-risk profile presented that increased number of deaths and the increased risk score were positive correlation (Figure 4D, 4J). In the TCGA cohort, we recognized that the area under curves (AUCs) in prognosticating 1-year, 2-year, and 3-year OS respectively were 0.757, 0.702, and 0.694 (Figure 4E). It is no difference with the results for the TCGA cohort, that AUC values for the 1-year, 2-year, and 3-year OS in the ICGC cohort were 0.744, 0.629, and 0.685, respectively (Figure 4K). In addition, in the TCGA-LIHC cohort, AUC values were significantly higher in BMs models than for age, sex, pathologic stage, and tumor stage (Figure 4F). Meanwhile, compared with the predictive power of age and gender, the BMs model exposed greater predictive power in the ICGC cohort (Figure 4L). The prognostic value of MMP1, ITGA2, P3H1, and CTSA in HCC are demonstrated using databases from TCGA-LIHC, ICGC, and GSE54236 HCC datasets (Supplementary Figures 1–3), which demonstrated that the four genes’ predictive value for HCC prognosis to a certain extent, though not as significant as the risk model overall. Furthermore, we utilized GSE54236 HCC datasets to validate our risk model and the results have significant statistical significance (Supplementary Figure 4). Interestingly, the risk model was verified to have significantly predicted value not only for HCC prognosis, but also for the tumor doubling time, a critical event for tumor progression.

Broadly speaking, a new model of risk score about HCC was created to efficiently estimate individual prognosis by multivariate Cox regression analysis, which was validated by ICGC queue and was compared with other effective predictive clinicopathological indicators.

### BMs model independence prognostic value, construction of nomograms and clinical correlation analysis

**Figure 5:**
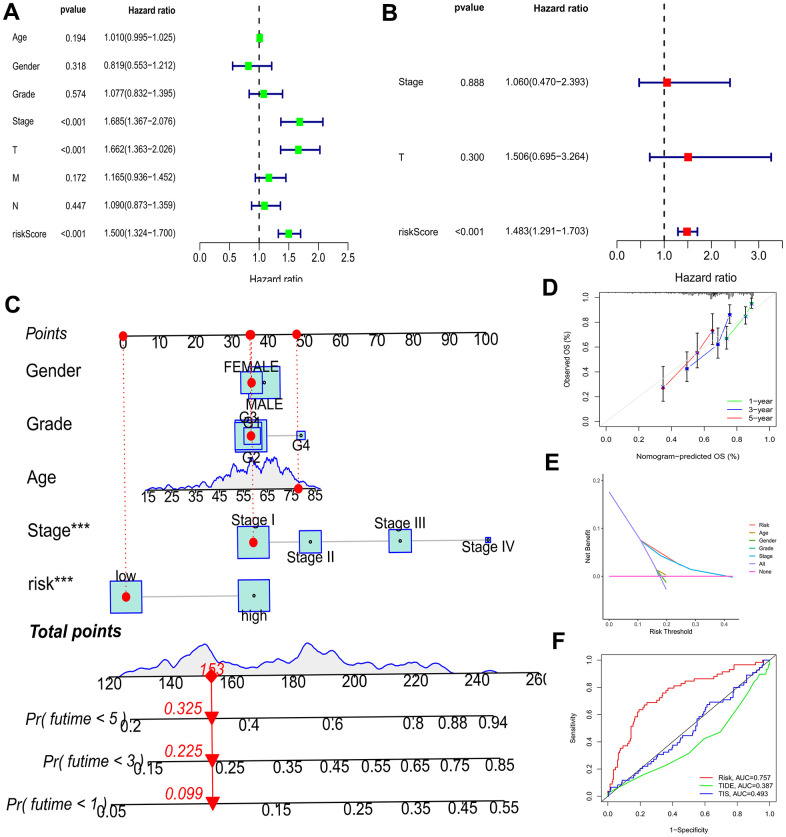
**Efficacy estimate of prognostic model and nomogram.** (**A**, **B**) Univariate Cox regression analysis of the model in the TCGA-LIHC cohort. (**C**) The nomogram consists of risk score and other clinicopathological parameters. (**D**) Calibration curves of the nomogram. (**E**) The decision curve analysis of the model in the TCGA-LIHC cohort. (**F**) AUC value of the BMs compared with other models in the TCGA cohort. *** p < 0.001.

**Figure 6:**
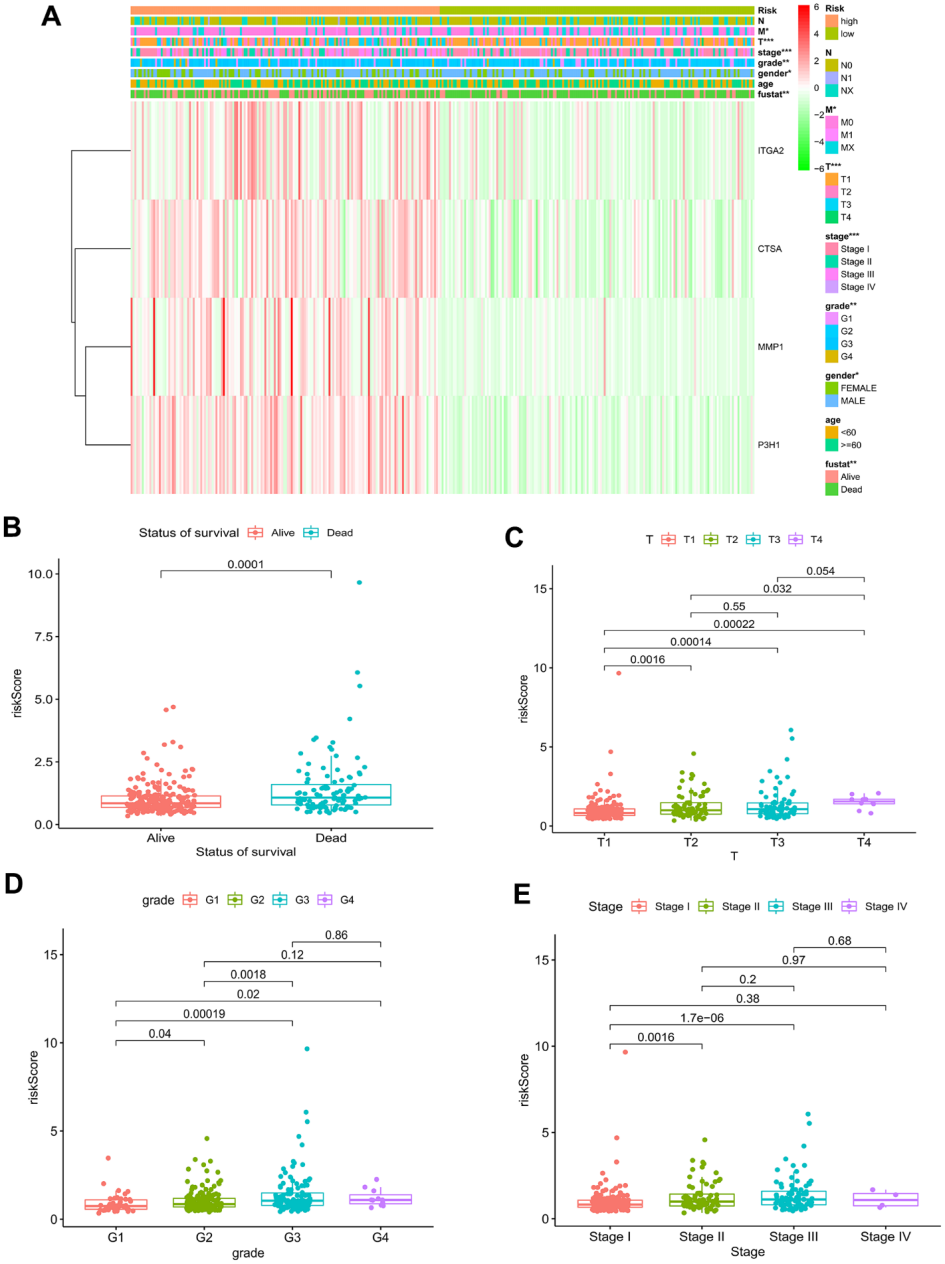
**Relationship of risk model and clinical characteristics.** (**A**) The heatmap of the model and clinical characteristics in the TCGA–LIHC cohort. Boxplot of risk score in HCC patients with different status of survival (**B**), T status (**C**), pathological grades (**D**), and tumor stages (**E**). * p < 0.05; ** p < 0.01; *** p < 0.001.

As depicted in Figure 5A, 5B, the TCGA-LIHC cohort revealed that BMs acted as an independent predictor of survival. This conclusion was drawn from the results obtained using both univariate and multivariate Cox regression models, indicating that BMs significantly influenced survival outcomes irrespective of other variables. Subsequently, nomograms of clinical adaptation were constructed by BMs models and other clinicopathological features, providing a visual method for prognosticating survival rates for the 1-year, 3-year, and 5-year time points for HCC (Figure 5C). Regarding predicting both short- and long-term survival, the nomogram has remarkable accuracy. The calibration plots of the column line plots revealed that the predictions of the column line plots and the actual observed probabilities were excellently coincident (Figure 5D). In addition, the net clinical benefit of BMs was higher than other clinicopathologic features in the TCGA-LIHC cohort, as indicated by decision curve analysis (Figure 5E). Significantly, compared with other models, AUC value of the BMs possessed superior advantage (Figure 5F). Moreover, in the TCGA-LIHC cohort, we found out risk scores were extremely related with T status, pathologic grade, and tumor stage (Figure 6, P < 0.05).

Collectively, the nomogram suffering BMs exhibited remarkable forecast of survival outcomes in the short and long run in patients diagnosed with HCC, which could boast prognostic value and assist in clinical management. And the decision curve analysis contributed to clinical decision and received better benefit.

### Analysis of tumor mutation burden and immune cell infiltration in different risk profiles

**Figure 7:**
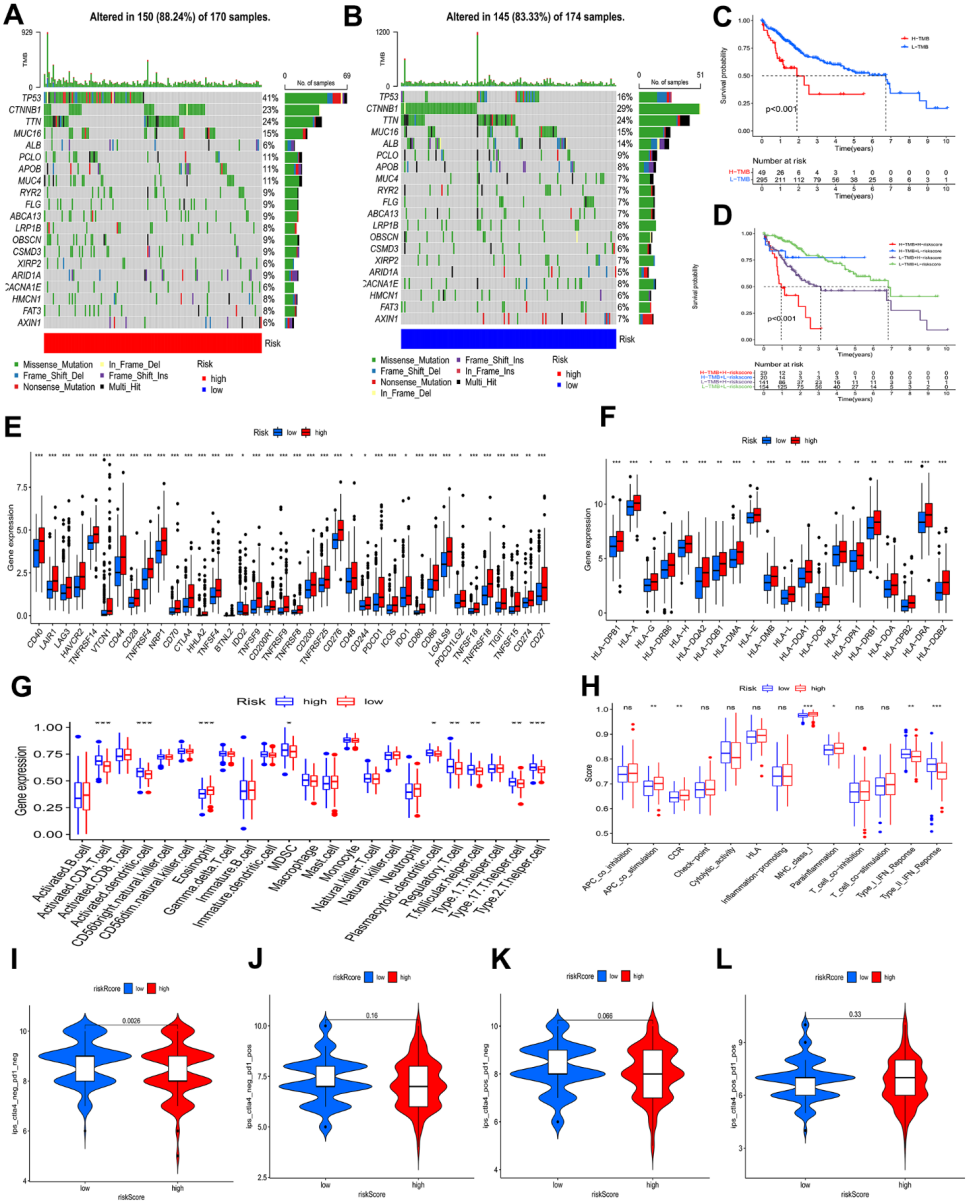
**Analysis of tumor mutation and immune cell infiltration.** (**A**, **B**) The waterfall plot showing the differences in somatic genomic mutation between high and low risk categories. (**C**, **D**) The Kaplan−Meier curve based on both TMB categories and the model for HCC patients. (**E**, **F**) Different risk categories expressed different levels of checkpoint genes and HLA genes. (**G**, **H**) Immune cell and immune function scores of different risk categories. Comparison of IPS between two risk categories. (**I**) CTLA4− PD1−, (**J**) CTLA4− PD1+, (**K**) CTLA4+ PD1−, and (**L**) CTLA4+ PD1+. ns p > 0.05; * p < 0.05; ** p < 0.01; *** p < 0.001.

The waterfall plots depicted variations in mutations of the top 20 genes among distinct risk profiles in patients with HCC, and higher mutation frequencies belonged to the patients at an elevated risk. (Figure 7A, 7B). TP53 was significantly more frequently mutated regarding the patients at an elevated risk than in the patients at a decreased risk, in contrast CTNNB1 was greatly more frequently mutated regarding the patients with a low-risk profile, mainly consisting of frameshift deletions and nonsense mutations. Tumor mutation burden (TMB), known as a nonsynonymous variant, demonstrates a strong association with the penetration of immune cells and the elicitation of immune responses [19]. Individuals with HCC were divided into a lower TMB profile and a higher TMB profile thanks to the optimal threshold. Kaplan-Meier curves demonstrated that patients with lower TMB had better events (Figure 7C). Given TMB may make a big difference in clinical experiments, we sought to investigate the combined impact of TMB and BMs. TMB and BMs exhibited synergistic effects on survival outcomes of HCC patients (Figure 7D).

To investigate the attributes of the tumor immune microenvironment in the two risk categories, we investigated the expression profiles of 38 immune checkpoint inhibitor (ICI) genes, 20 HLA genes, and the frequency of infiltrating immune cell populations within the tumor. In the high-risk category (Figure 7E), we observed a substantial upregulation of immune checkpoint genes, indicative of heightened immunosuppressive signaling. Additionally, HLA-associated genes were prominently overexpressed in the same high-risk profile (Figure 7F), suggesting a potential role in modulating antigen presentation and immune recognition. Moreover, leveraging the “ssGSEA” algorithm, we quantified the abundance of penetration of immune cells, unveiling a significant heterogeneity in the immune cell landscape between the two risk profiles, reflecting differential immune cell infiltration patterns and potentially distinct immune responses. Some immune cells levels were remarkably higher in the patients with a high-risk profile, which involved in activated CD4 T cells, activated dendritic cells, myeloid-derived suppressor cells (MDSCs), plasmacytoid dendritic cells, regulatory T cells, T follicular helper cells, type 17 T helper cells, and type 2 T helper cells (Figure 7G), as found by the results of ssGSEA. Type I interferon (IFN) responses and type II IFN responses had an enrichment in the group of low risk, and antigen presenting cell (APC) costimulation, Chemokine receptors (CCR), major histocompatibility complex (MHC) class I, and para-inflammation showed enrichment in the group of high risk (Figure 7H).

The BMs Models predicted tumor immune microenvironment (TIME), with the patients at an elevated risk showing higher expression of 38 ICI genes, 20 HLA genes, and increased tumor-infiltrating immune cells. The high-risk profile also exhibited enrichment in immune processes like APC costimulation, CCR signaling, MHC class I pathway activation, and para-inflammation, potentially explaining the significant difference in prognosis between the subgroups.

### The role of BMs in immunotherapy

An IPS analysis was conducted to further test the BMs model’s ability to prognosticate the ICI efficacy and to determine immunotherapy sensitivity in HCC patients separately. A significantly higher IPS-CTLA4-neg-PD1-neg score, in the low-risk profile than in the high-risk profile, as shown in Figure 7I–7L, demonstrated that the immunogenic phenotype is stronger in the patients with a low-risk profile and therefore they may acquire benefits from immunotherapy.

**Figure 8:**
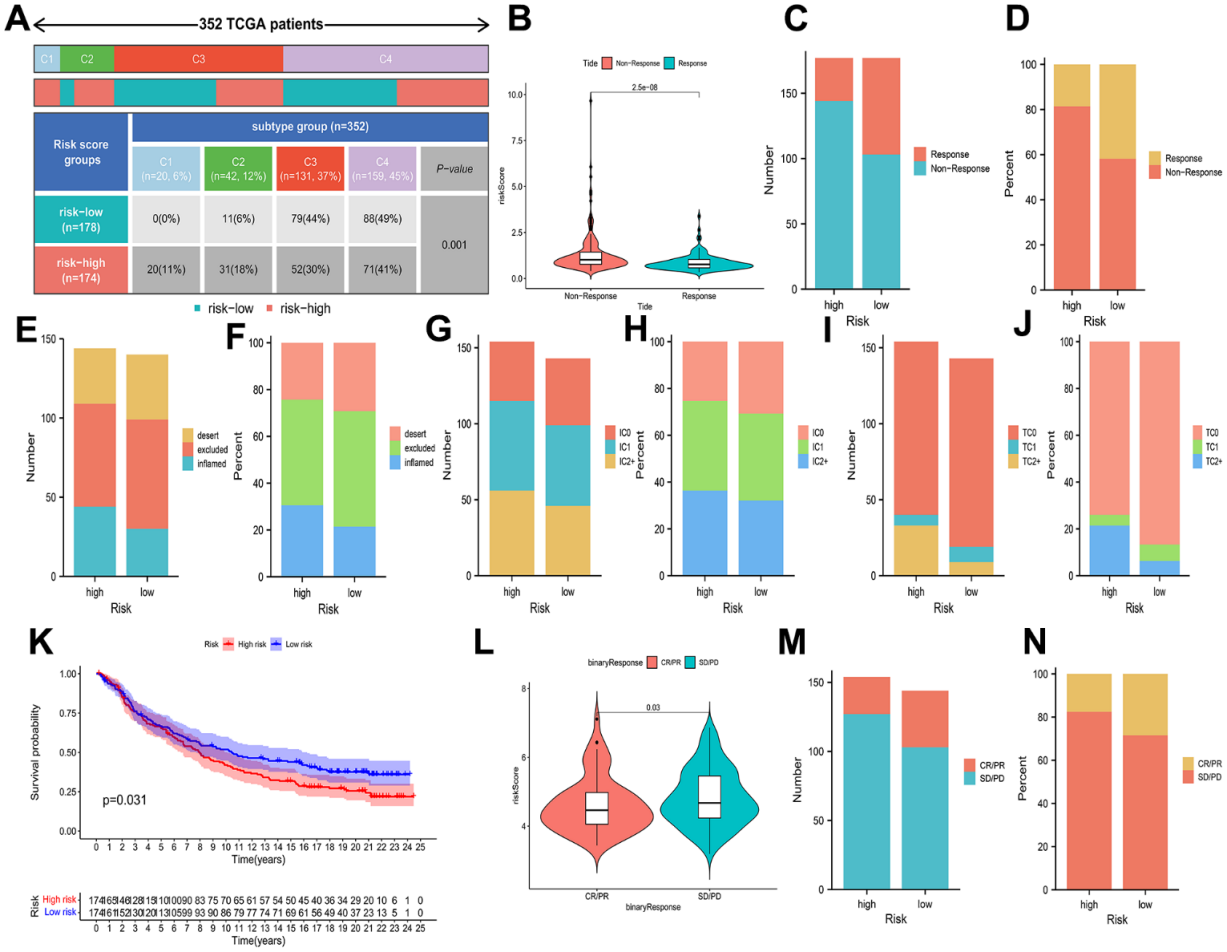
**Associations of prognostic risk model and HCC immunotherapy.** (**A**) A comparison of immune subtypes between different risk categories. (**B**) A comparison of risk scores between two response categories. (**C**–**J**) Stacked bar plot of rates of response, inflammatory immune subtypes, tumor-infiltrating immune cells, and PD-L1-expressing tumor tissue samples. (**K**) Kaplan-Meier curves of different risk categories. (**L**) A comparison of risk scores between the CR/PR category and stable disease (SD)/progressive disease (PD) category. (**M**, **N**) Stacked bar plot of CR/PR and SD/PD.

In addition, the ratio of HCC immune subtypes in the different risk groups was compared. It was a statistically significant difference that the immunophenotype analysis presented by comparing the different groups (Figure 8A). It was further evaluated whether BMs could be utilized as a predictive factor of immunotherapy in HCC individuals. The patients with a low-risk profile exhibited increased rates of remission than the individuals with a high-risk profile (Figure 8B–8D). In the IMvigor210 cohort, 348 patients were split into high-risk and low-risk profiles, with metastatic uroepithelial carcinoma after the treatment of anti-PD-L1 drugs. Between the two profiles, statistically remarkable differences were observed in the proportions of inflammatory immune subtypes, tumor-infiltrating immune cells, and PD-L1-expressing tumor tissue samples (Figure 8E–8J). Kaplan-Meier curves presented that individuals with low-risk scores had better prognosis (Figure 8K). In comparison to the patients at an elevated risk, the patients at a decreased risk possessed a higher percentage of complete remission (CR)/partial remission (PR) (Figure 8L–8N).

Consequently, the response to immunotherapy varied between the two risk profiles defined by BMs, with patients in the low-risk category potentially exhibiting heightened sensitivity to immunotherapy, leading to more favorable clinical outcomes.

### Drug sensitivity of BMs in HCC

**Figure 9:**
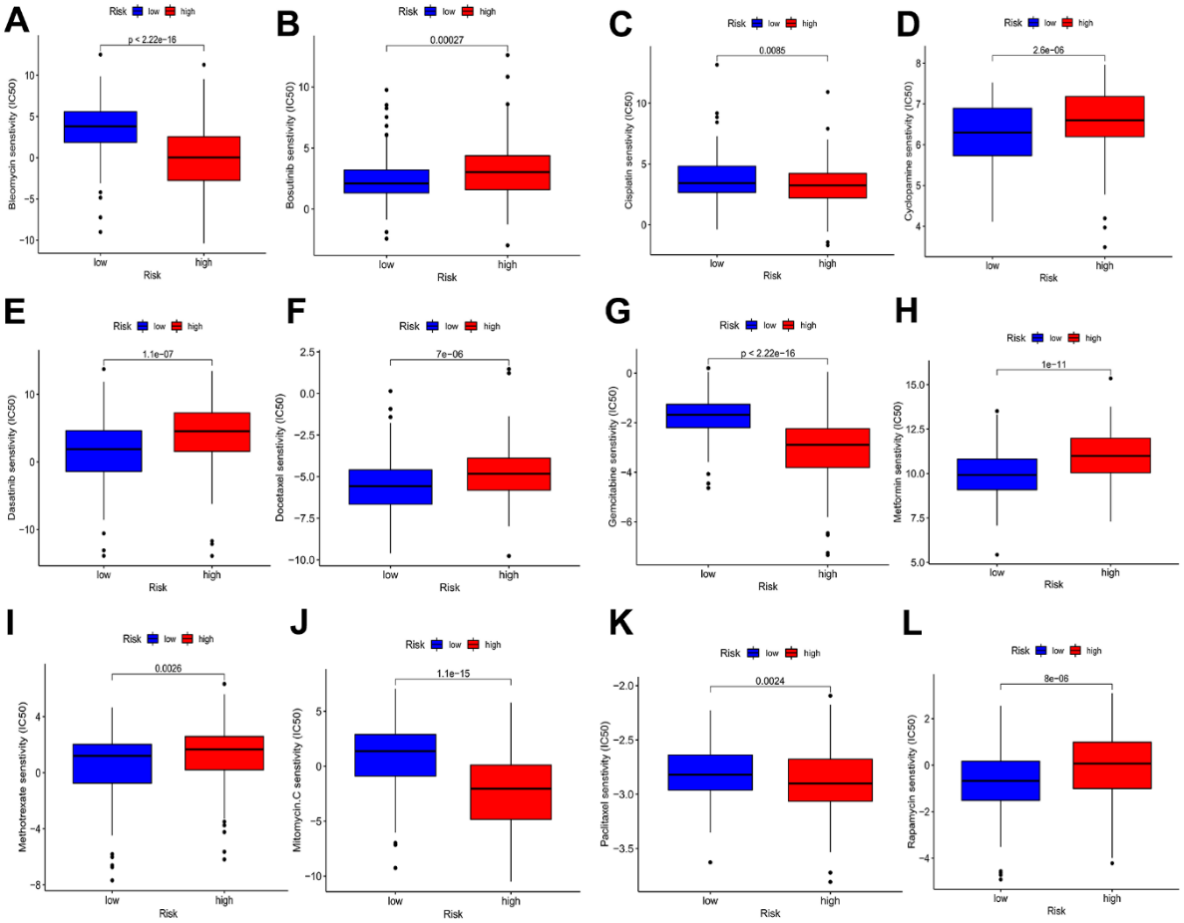
**Drug sensitivity analyses among high- and low-risk categories.** (**A**) Estimated IC_50_ of bleomycin in the elevated-risk category were lower than in the decreased-risk category. (**B**) Estimated IC_50_ of bosutinib in the elevated-risk category were higher than in the decreased-risk category. (**C**) Estimated IC_50_ of cisplatin in the elevated-risk category were lower than in the decreased-risk category. (**D**, **E**, **F**) Estimated IC_50_ of cyclopamine, dasatinib and docetaxel in the elevated-risk category were higher than in the decreased-risk category. (**G**) Estimated IC_50_ of gemcitabine in the elevated-risk category were lower than in the decreased-risk category. (**H**, **I**) Estimated IC_50_ of metformin and methotrexate in the elevated-risk category were higher than in the decreased-risk category. (**J**, **K**) Estimated IC_50_ of mitomycin C and paclitaxel in the elevated-risk category were lower than in the decreased-risk category. (**L**) Estimated IC_50_ of rapamycin in the elevated-risk category were higher than in the decreased-risk category.

With the aim of investigating the possible use of BMs in HCC personalized treatment and further facilitating the clinical value of BMs for dealing with HCC, we evaluated several chemotherapeutic agents for their IC_50_ values between different risk profiles. The IC_50_ value demonstrated an inverse relationship with the responsiveness of the chemotherapy drugs. The drug sensitivity demonstrated that the IC_50_ values of bleomycin, cisplatin, gemcitabine, mitomycin C, and paclitaxel in the elevated-risk category exhibited lower IC_50_ values than those in the decreased-risk category, implying that high risk individuals is likely to receive more beneficial effects from the above chemotherapies, while bosutinib, cyclopamine, dasatinib, docetaxel, metformin, methotrexate and rapamycin in the low-risk profile were lower, indicating that the decreased-risk category may benefit more from the above chemotherapies (Figure 9A–9L).

Based on these results, the risk score has the potential to guide patients towards receiving more tailored chemotherapies.

### The differential expression of BMs model in cell lines of HCC

**Figure 10:**
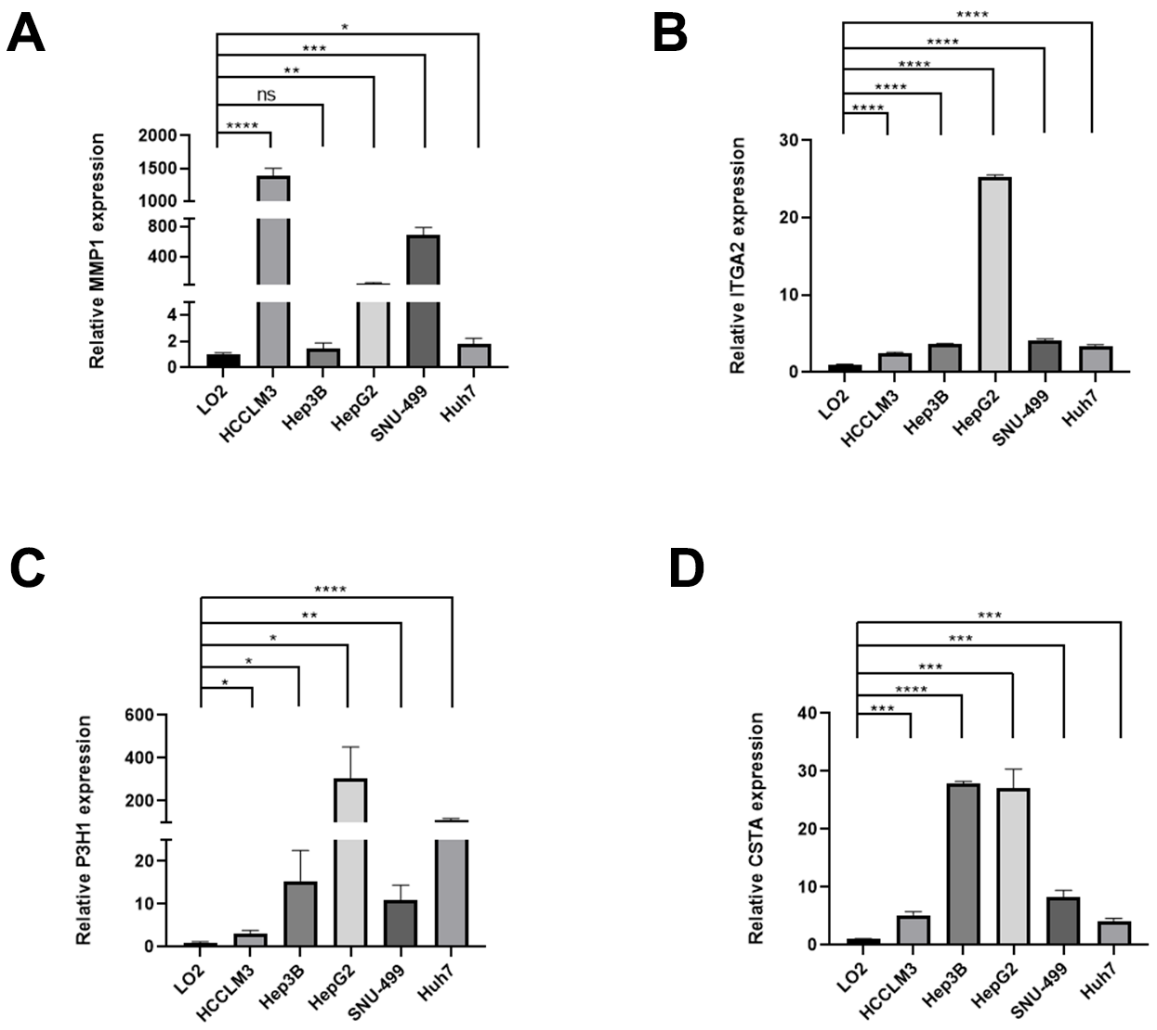
**The expression differences of normal liver and HCC cell lines.** The expression differences of MMP1 (**A**), ITGA2 (**B**), P3H1 (**C**), and CTSA (**D**) in normal liver and HCC cell lines.

In the preliminary work, a risk model based on BMs-related gene was constructed. Now, we further verified these results *in vitro*, 4 genes of the model were subjected to qRT-PCR. Compared with LO2 cells, the expression levels of 4 genes in five types of HCC cells were significantly differential (Figure 10A–10D).

## DISCUSSION

Considered as a major global health burden, liver cancer is expected to affect more than one million people by 2025 [20]. Generally, cancer of the liver develops from chronic hepatitis with infiltration by various immune cells. Its aggressiveness, heterogeneity and usually advanced stage of diagnosis contribute to its poor prognosis [21]. Currently, western immune checkpoint inhibitors offer great hope for patients with HCC. Therefore, new and reliable models and a better understanding of the link between models and cancer immunity are essential for patients with HCC.

Greater understanding of BMs has helped to make them a hot topic of research over the past years. However, no studies have investigated the predictive value of BMs in cancer. Taking advantage of the genetic characteristics of genes associated with BMs, our novel model was developed for predicting survival and treatment decisions in HCC patients.

In our research, amount to 222 BMGs were first acquired from the available literature. Then, we obtained the tumor-associated genes of HCC by WGCNA. Identification of 66 BMGs intersected with tumor-associated genes for further study. When employing GO and KEGG enrichment analysis, we determined these genes enriched in ECM-receptor interactions, adherent spots, and the PI3K-Akt signaling pathway. The prognostic significance of these genes was evaluated, and subsequently, we recognized 20 genes associated with prognosis through a univariate Cox regression analysis. Utilizing a multivariate Cox regression analysis, we constructed a BMs correlation model and validated its performance using the ICGC cohort. Risk Score = 0.238*MMP1 + 0.212*ITGA2 + 0.400*P3H1 + 0.240*CTSA is the result of this algorithm. According to their median risk score, patients with HCC in the TCGA and ICGC cohorts were separated into elevated-risk and decreased-risk categories. Higher risk scores were corresponding to poorer individuals results, as determined by Kaplan-Meier curves. The ROC curves demonstrated the model’s accuracy. The BMs model was a predictor of survival in univariate and multivariate Cox regression investigations for the TCGA-LIHC cohort. As a result, we additionally implemented the risk model to determine the connection between tumor mutational load and immune cell infiltration. The waterfall plot illustrates that the high-risk patient profile manifested a greater prevalence of mutations in the top 20 genes. The elevated-risk category possessed significantly more TP53 mutations than the decreased-risk category, whereas the low-risk profile featured significantly more CTNNB1 mutations. The expression of immune checkpoint genes appeared to be greater in the high-risk profile. 20 HLA genes and 38 ICI genes encountered high levels of expression in the low-risk individuals. More intriguingly, IPS-CTLA4-neg-PD1-neg scores in the profile with a lower risk and the profile with a higher risk significantly differed, indicating a more immunogenic phenotype in those in the low-risk category. In contrast, the proportion of immune cells between the two categories. However, several limitations of our study do exist. First, more thorough research should be done to confirm the model’s predictive utility in a clinical environment. As our research was only based on retrospective data from public databases, prospective studies will be required in the future to substantiate our discoveries.

MMP1, ITGA2, P3H1 and CTSA were recruited in this BMs risk model of HCC. As we know, MMP1 is identified as a matrix metalloproteinase capable of degrading various components of the extracellular matrix (ECM), facilitating tumor cell invasion and metastasis. It promotes ECM remodeling, assisting tumor cells in breaching the basement membrane and surrounding tissue, which in turn supports angiogenesis and tumor growth [22]. ITGA2, a member of the integrin family, plays a role in cell-cell and cell-matrix interactions. Wang et al. reported ITGA2 may promote HCC progression by activating cell adhesion, migration, and survival pathways, such as FAK/PI3K/Akt pathway and modulating the interactions between tumor cells and their microenvironment [23]. The specific role of P3H1 in HCC is not as well elucidated as MMP1 or ITGA2, which deserved the further explored in the future [24]. As reported, CTSA is overexpressed in various types of cancer and is linked to poor clinical outcomes. The high protein level of CTSA was significantly correlated to the poor clinicopathological parameters, such as TNM stage, vascular invasion, tumor recurrence, and patient death of HCC [25]. Overall, further experiments need to be carried out to better understand the mechanism of these genes in HCC.

In conclusion, we identified a BMs-related model for HCC patients in the present investigation. What’s more, the same model was validated to forecast HCC patients’ OS and demonstrated excellent function of prediction. We also investigated disparities in immunotherapy response and sensitivity to chemotherapeutic medications among BMs risk categories. Our study contributes to the advancement of understanding immune infiltration characteristics and innovative approaches for personalized treatment.

## MATERIALS AND METHODS

### Data source

In this work, we obtained 222 BMGs from existing literature [26]. Additionally, we downloaded clinical data, genomic mutation profiles, and RNA-seq expression data regarding HCC patients from the Cancer Genome Atlas (TCGA) database. Additionally, we leveraged the gene expression data from the IMvigor210 dataset and the corresponding clinical information of HCC patients undergoing immune checkpoint inhibitor therapy. Utilizing the “CoreBiologies” R package, we corroborated that our risk model had the capability to anticipate the response of HCC patients to immunotherapy.

### WGCNA

WGCNA can effectively identify gene set modules that display a strong association. The parameter setting and steps in WGCNA analysis were as followed. Initialization and data preparation: utilization of the WGCNA and limma packages, specifying samples. Data pre-processing: numerical conversion of gene expression data, application of avereps function for log2 transformation, and filtering genes with a standard deviation less than 0.5. Sample clustering: hierarchical clustering based on expression data, using average linkage method, and detection of outliers through sample dendrogram. Network construction: selection of an appropriate soft threshold (power) with pickSoftThreshold, construction of adjacency and TOM matrices, followed by gene clustering. Module identification: dynamic tree cutting for gene module identification, calculation of module eigengenes (MEs), and module similarity clustering. Gene significance and module membership: calculation of gene significance for clinical traits and module membership, with visualization through Risk Score Triad Diagrams for each clinical feature and module.

In this study, we used WGCNA to calculate correlation weights of coexpression and identify coexpressed genes associated with tumors [27]. Rather than solely relying on differential gene expression, WGCNA allowed us to pinpoint relevant gene sets and conduct rigorous association analyses with phenotypic traits. This approach effectively shifted the challenge of correcting for multiple hypothesis testing from handling correlations between thousands of individual genes and phenotypes to examining the associations of a few gene sets with the phenotypes. A scale-free network was generated by specifying the optimal soft threshold power. To continue, we computed gene dendrograms and modules utilizing the 50-min cluster size based on the topological overlap matrix TOM dissimilarity. We identified the most significant module associated with HCC and used it for further analysis. We found the intersection genes of the module genes that related with HCC and BMGs by Venn plot.

### Functional and pathway enrichment analysis

To assess the characteristic functional attributes of the two risk groups, we conducted an analysis of the shared genes between the two groups and subsequently annotated them using the “clusterProfiler” R package with information from the Gene Ontology (GO) and Kyoto Encyclopedia of Genes and Genomes (KEGG) databases [28].

### Prognostic, correlation and mutation analysis

The prognosis-related BMGs were checked out using univariate Cox proportional hazard regression. The prognosis-related BMGs correlation pheatmap is displayed by the R software package. Furthermore, we utilized the “ggplot” R package to explore the relationship between BMGs and the abundance of immune cells within the tissue. We established a significance threshold of P<0.05 for statistical significance. The R package “maftools” then showed the mutation status of prognosis-related BMGs [29]. In addition, the frequency of copy number variations (CNVs) in prognosis-related BMGs was calculated. The chromosome positions of these genes were visualized through “RCircos” software package.

### Construction and validation of BMs model

Using multivariate Cox regression, we developed a robust prognostic model for BMGs. External validation was performed on the International Cancer Genome Consortium (ICGC) cohort. Patients were categorized into high- and low-risk profiles based on the median BMGs expression score. Kaplan-Meier curves were employed for precise comparison of overall survival (OS) between the two risk profiles, offering critical insights for tailored treatment approaches in HCC patients. The classification results were validated by using principal component analysis (PCA) and t-distribution random neighborhood embedding (t-SNE) to obtain low-dimensional clustering distributions from high-dimensional gene sets. “survivalROC” and “timeROC” R packages were used to quantify the prognostic model’s forecast value. To better understand the prognostic nomogram using the “regplot” package in this article, we showed the constructed details. Cox proportional hazards model construction: survival data were analyzed using the Cox proportional hazards model, with risk scores and clinical variables serving as covariates. Nomogram generation: utilizing the Cox model’s results, nomograms were created via the regplot function to visualize the impact of risk scores on survival probabilities. Calibration curve generation: calibration curves were produced using the calibrate function, comparing the predicted survival probabilities from nomograms against observed survival rates to evaluate the model’s predictive performance. This study involved constructing a prognostic nomogram using the “regplot” package, incorporating clinical factors and gene signatures to estimate 1-, 3-, and 5-year OS probabilities. To assess the predictive capacity of column line graphs, 3-year and 5-year calibration curves were generated. Furthermore, decision curve analysis was utilized to assess the clinical applicability of the risk score model by computing the net benefit across different risk thresholds [30]. Additionally, we also compared 1-year receiver operating characteristic (ROC) value of the model with other models.

### Exploration of immune infiltration

Tumor microenvironment (TME) cells, a crucial component of tumor tissue, hold significant clinicopathological importance in prognosticating outcomes and treatment effectiveness, supported by emerging evidence. Further research was completed on the expression of human leukocyte antigen (HLA) genes and common immunological checkpoints in various clusters. To compare the immune infiltration and immunological function of each group, a Wilcoxon test with two samples was performed. The single-sample gene set enrichment analysis (ssGSEA) algorithm was applied for immunological inverse fold product analysis of 23 immune cell type gene sets, enabling exploration of TME infiltration in high-risk and low-risk profiles [31]. The 23 different TME cell types and the risk category were correlated via Spearman correlation analysis.

### Estimation of immunotherapeutic response prediction

The liver hepatocellular carcinoma (LIHC) project of The Cancer Immunome Atlas is where the immunophenoscore (IPS) of HCC samples was developed. CTLA4 and PD-1 blockers are part of this project’s method for forecasting the effectiveness of immunotherapies [31]. Moreover, the immune subtypes of HCC in the TCGA database have been described in a previous study [32] and we analyzed immune subtype proportions between the two risk groups based on UCSC-Xena database while using the “RColorBrewer” package to provide color schemes and create visualizations of immune clustering results. Additionally, we confirmed the link between immune checkpoint inhibitors and HCC risk markers thanks to the IMvigor210 dataset.

### Chemotherapeutic drug sensitivity analysis for different risk groups

We conducted a pharmacore sensitivity analysis using the “prophecy” and “ggplot2” packages and compared the sensitivity of various chemotherapeutic agents between the groups of high risk and groups of low risk utilizing the Wilcoxon signed-rank test in the absence of official biomarkers to accurately predict the reaction of chemotherapeutic agents in patients with HCC. The half-maximal inhibitory concentrations (IC_50_) of different chemotherapeutic drugs were compared between HCC high-risk category and low-risk category [33].

### Cell culture

Human hepatic cell line LO2 and five human HCC cell lines (HCCLM3, Hep3B, HepG2, SNU-499 and Huh7 cells) were flashed frozen in liquid nitrogen with 2 ml tubes and stored at −80° C. All cell lines were cultured in 10% fetal bovine serum (FBS; Invitrogen, Carlsbad, CA, USA) in DMEM medium. All cell lines grew in a humid environment of 37° C, 5% CO_2_, 99% relative humidity and did not contain antibiotics. The cells were subcultured at a ratio of 1:2 or 1:3 when they reached 80% confluence.

### RNA extraction and quantitative real-time PCR (qRT-PCR)

TRIzol reagent kit (Invitrogen) was used to extract the total RNA from logarithmic growth cells. cDNA was synthesized using PrimeScript RT reagent kit (Takara Biotechnology, Dalian, China). QRT-PCR analysis was conducted using TB Green Premix Ex Taq II kit (Takara Biotechnology, Dalian, China) according to the instructions, three replicates were set in each well. All operations were carried out on ice. The 2^−ΔΔCt^ method was used for qRT-PCR analysis. The primers were designed using the Primer Bank (https://pga.mgh.harvard.edu/primerbank/) and the NCBI primer-BLAST tool (https://www.ncbi.nlm.nih.gov/). The sequences of primers were shown as Table 1, GAPDH was used as reference genes.

**Table 1:** Sequences of primers of BMs model and GAPDH.

**Gene**	**Sequences of primers**
GAPDH	Forward Primer: ACAACTTTGGTATCGTGGAAGG
	Reverse Primer: GCCATCACGCCACAGTTTC
MMP1	Forward Primer: AAAATTACACGCCAGATTTGCC
	Reverse Primer: GGTGTGACATTACTCCAGAGTTG
ITGA2	Forward Primer: CCTACAATGTTGGTCTCCCAGA
	Reverse Primer: AGTAACCAGTTGCCTTTTGGATT
P3H1	Forward primer: CAGCTCGAGCGGGACAG
	Reverse primer: AGGTCCATCTCTTCTGGGCT
CTSA	Forward Primer: GTCGCCCAGAGCAATTTTGAG
	Reverse Primer: TCTCCCCGGTCAGGAAAAGTT

### Statistical analysis

Spearman’s rank correlation was employed to investigate the relationship between TIP scores and immune traits. Kaplan-Meier analyses and log-rank tests, facilitated by the “survival” package, assessed survival outcomes. Additionally, the Pearson Chi-square test explored the relationship between the TIPRGPI group and clinicopathological variables. For univariate and multivariate determination of independent prognostic indicators, the “survival” package was studied. The optimal threshold for survival analysis was determined using the “survminer” R package. All statistical analyses were conducted using R software (version 3.6.1). p < 0.05 was accepted as indicative of significant differences if there is no other case.

### Data availability statement

The original contributions presented in the study are included in the article. Further inquiries can be directed to the corresponding author.

## Supplementary Material

Supplementary Figures

Supplementary Table 1
