# Supplementary material for: Comprehensive evaluation of genes related to basement membrane in hepatocellular carcinoma
Source: Aging (Albany NY). 2024 Jun 12;16(11):10108–31. doi: 10.18632/aging.205923 (PMC11210257; doi:10.18632/aging.205923)
Supplement: Supplementary Figures [file aging-16-205923-s001.pdf]

## SUPPLEMENTARY FIGURES

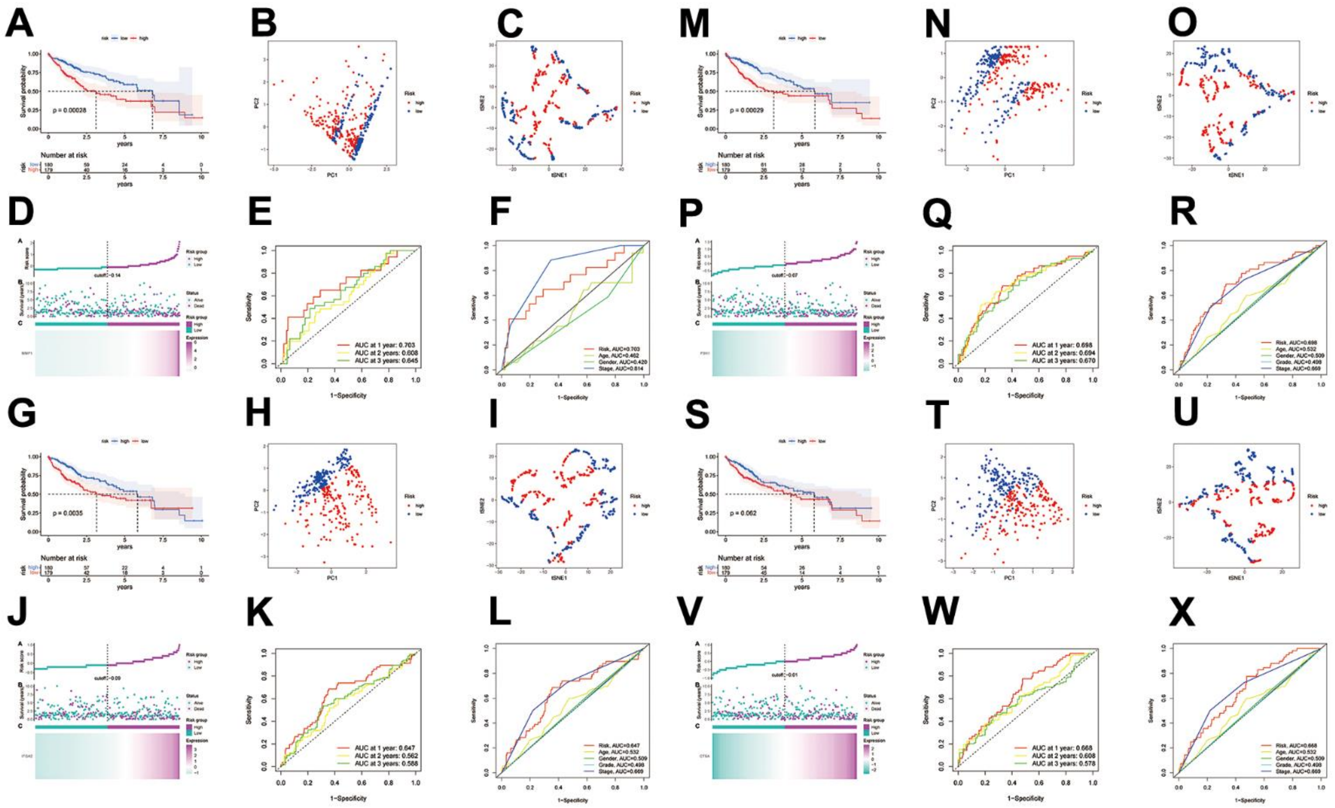


**Supplementary Figure 1. The TCGA database was utilized to individually validate the prognostic value of MMP1, ITGA2, P3H1, and CTSA in hepatocellular carcinoma.** (**A**) Kaplan-Meier curve of HCC patients in the MMP1. (**B**, **C**) PCA and t-SNE analysis showing a remarkable difference in transcriptomes between the two risk categories in the MMP1. (**D**) Risk Score Triad Diagrams showing the risk score distribution and patient survival status in the MMP1. (**E**) ROC curves to predict the sensitivity and specificity of 1-, 2-, 3-year survival according the risk score in the MMP1. (**F**) Clinical ROC analysis in the MMP1. (**G**) Kaplan-Meier curve of HCC patients in the ITGA2. (**H**, **I**) PCA and t-SNE analysis showing a remarkable difference in transcriptomes between the two risk categories in the ITGA2. (**J**) Risk Score Triad Diagrams showing the risk score distribution and patient survival status in the ITGA2. (**K**) ROC curves to predict the sensitivity and specificity of 1-, 2-, 3-year survival according the risk score in the ITGA2. (**L**) Clinical ROC analysis in the ITGA2. (**M**) Kaplan-Meier curve of HCC patients in the P3H1. (**N**, **O**) PCA and t-SNE analysis showing a remarkable difference in transcriptomes between the two risk categories in the P3H1. (**P**) Risk Score Triad Diagrams showing the risk score distribution and patient survival status in the P3H1. (**Q**) ROC curves to predict the sensitivity and specificity of 1-, 2-, 3-year survival according the risk score in the P3H1. (**R**) Clinical ROC analysis in the P3H1. (**S**) Kaplan-Meier curve of HCC patients in the CTSA. (**T**, **U**) PCA and t-SNE analysis showing a remarkable difference in transcriptomes between the two risk categories in the CTSA. (**V**) Risk Score Triad Diagrams showing the risk score distribution and patient survival status in the CTSA. (**W**) ROC curves to predict the sensitivity and specificity of 1-, 2-, 3-year survival according the risk score in the CTSA. (**X**) Clinical ROC analysis in the CTSA.

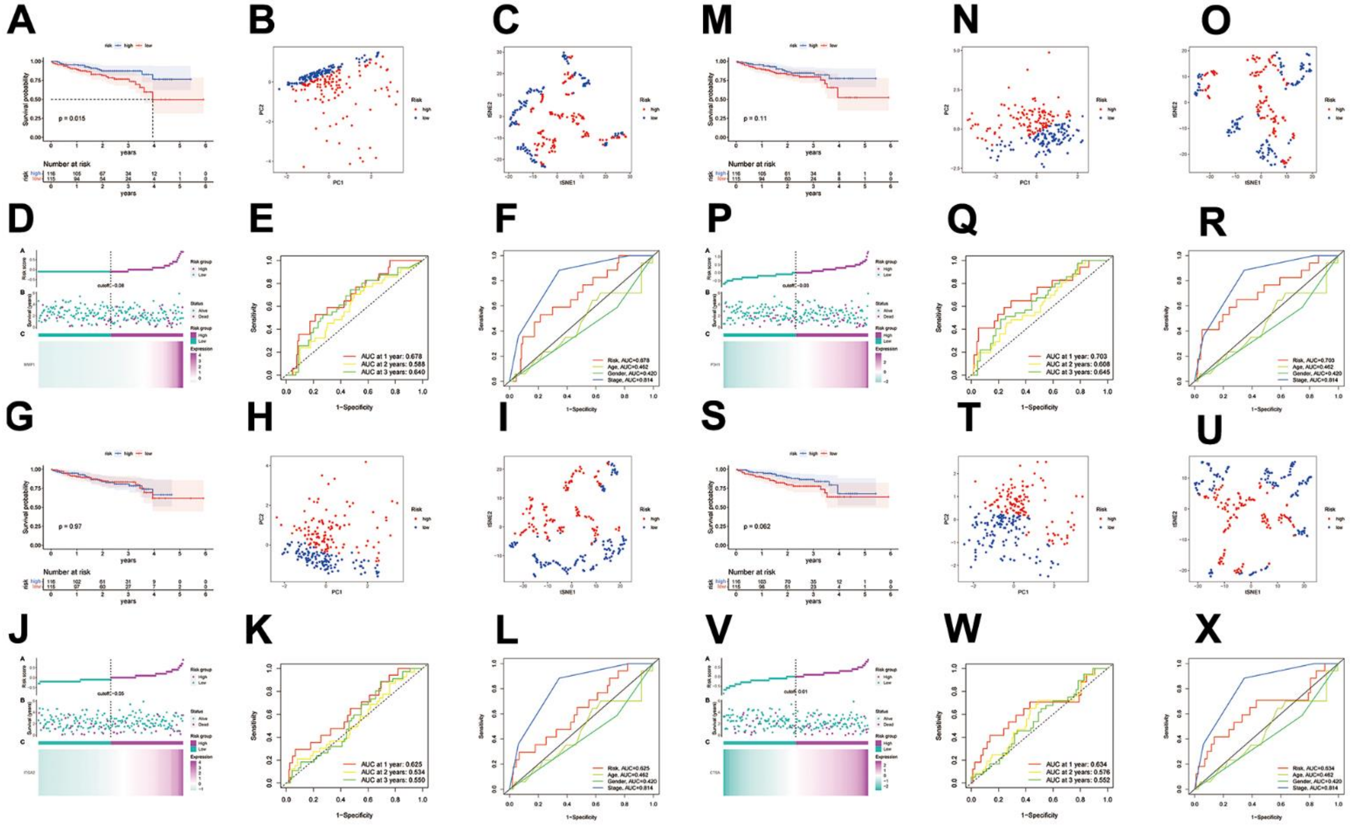


**Supplementary Figure 2. The ICGC database was utilized to individually validate the prognostic value of MMP1, ITGA2, P3H1, and CTSA in hepatocellular carcinoma.** (**A**) Kaplan-Meier curve of HCC patients in the MMP1. (**B**, **C**) PCA and t-SNE analysis showing a remarkable difference in transcriptomes between the two risk categories in the MMP1. (**D**) Risk Score Triad Diagrams showing the risk score distribution and patient survival status in the MMP1. (**E**) ROC curves to predict the sensitivity and specificity of 1-, 2-, 3-year survival according the risk score in the MMP1. (**F**) Clinical ROC analysis in the MMP1. (**G**) Kaplan-Meier curve of HCC patients in the ITGA2. (**H**, **I**) PCA and t-SNE analysis showing a remarkable difference in transcriptomes between the two risk categories in the ITGA2. (**J**) Risk Score Triad Diagrams showing the risk score distribution and patient survival status in the ITGA2. (**K**) ROC curves to predict the sensitivity and specificity of 1-, 2-, 3-year survival according the risk score in the ITGA2. (**L**) Clinical ROC analysis in the ITGA2. (**M**) Kaplan-Meier curve of HCC patients in the P3H1. (**N**, **O**) PCA and t-SNE analysis showing a remarkable difference in transcriptomes between the two risk categories in the P3H1. (**P**) Risk Score Triad Diagrams showing the risk score distribution and patient survival status in the P3H1. (**Q**) ROC curves to predict the sensitivity and specificity of 1-, 2-, 3-year survival according the risk score in the P3H1. (**R**) Clinical ROC analysis in the P3H1. (**S**) Kaplan-Meier curve of HCC patients in the CTSA. (**T**, **U**) PCA and t-SNE analysis showing a remarkable difference in transcriptomes between the two risk categories in the CTSA. (**V**) Risk Score Triad Diagrams showing the risk score distribution and patient survival status in the CTSA. (**W**) ROC curves to predict the sensitivity and specificity of 1-, 2-, 3-year survival according the risk score in the CTSA. (**X**) Clinical ROC analysis in the CTSA.

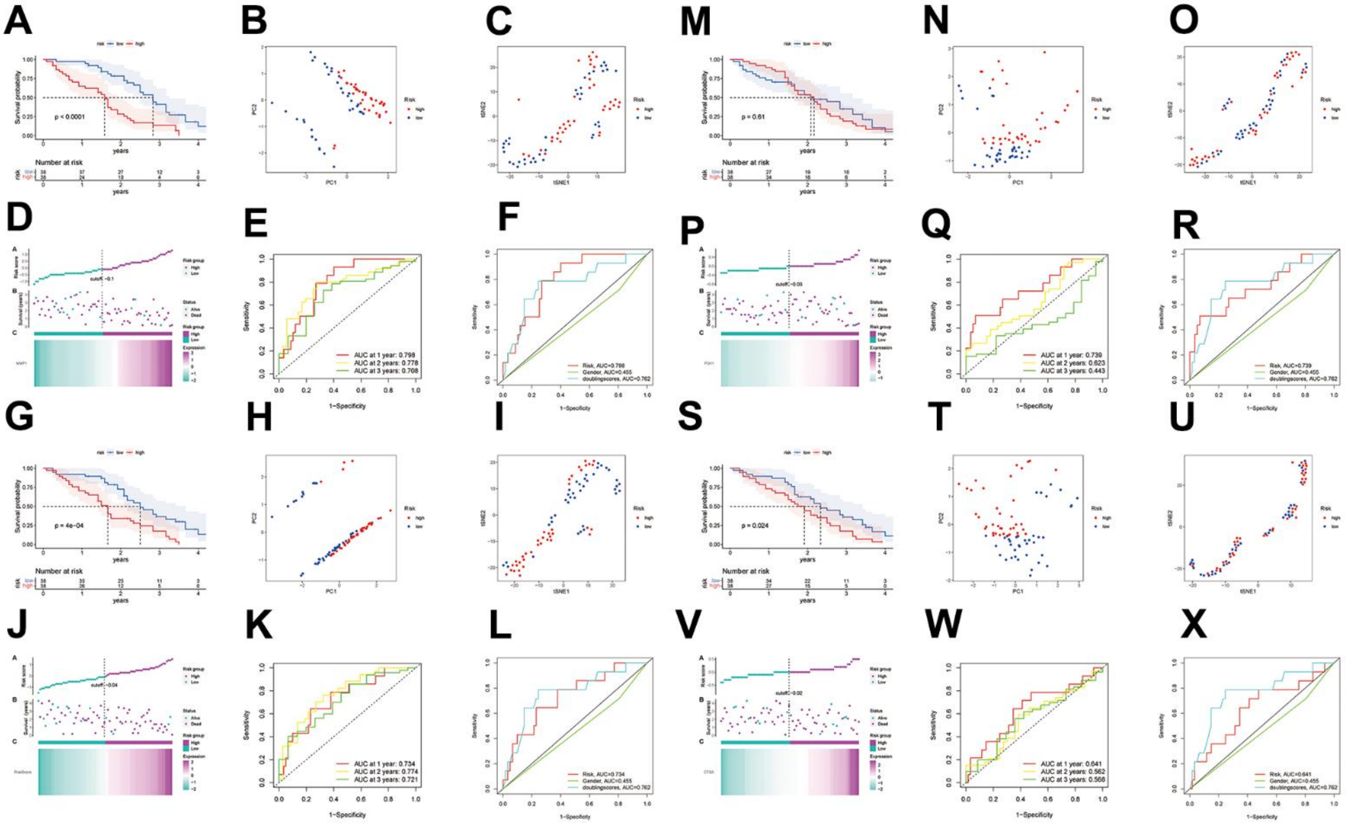


**Supplementary Figure 3. The GEO database was utilized to individually validate the prognostic value of MMP1, ITGA2, P3H1, and CTSA in hepatocellular carcinoma.** (**A**) Kaplan-Meier curve of HCC patients in the MMP1. (**B**, **C**) PCA and t-SNE analysis showing a remarkable difference in transcriptomes between the two risk categories in the MMP1. (**D**) Risk Score Triad Diagrams showing the risk score distribution and patient survival status in the MMP1. (**E**) ROC curves to predict the sensitivity and specificity of 1-, 2-, 3-year survival according the risk score in the MMP1. (**F**) Clinical ROC analysis in the MMP1. (**G**) Kaplan-Meier curve of HCC patients in the ITGA2. (**H**, **I**) PCA and t-SNE analysis showing a remarkable difference in transcriptomes between the two risk categories in the ITGA2. (**J**) Risk Score Triad Diagrams showing the risk score distribution and patient survival status in the ITGA2. (**K**) ROC curves to predict the sensitivity and specificity of 1-, 2-, 3-year survival according the risk score in the ITGA2. (**L**) Clinical ROC analysis in the ITGA2. (**M**) Kaplan-Meier curve of HCC patients in the P3H1. (**N**, **O**) PCA and t-SNE analysis showing a remarkable difference in transcriptomes between the two risk categories in the P3H1. (**P**) Risk Score Triad Diagrams showing the risk score distribution and patient survival status in the P3H1. (**Q**) ROC curves to predict the sensitivity and specificity of 1-, 2-, 3-year survival according the risk score in the P3H1. (**R**) Clinical ROC analysis in the P3H1. (**S**) Kaplan-Meier curve of HCC patients in the CTSA. (**T**, **U**) PCA and t-SNE analysis showing a remarkable difference in transcriptomes between the two risk categories in the CTSA. (**V**) Risk Score Triad Diagrams showing the risk score distribution and patient survival status in the CTSA. (**W**) ROC curves to predict the sensitivity and specificity of 1-, 2-, 3-year survival according the risk score in the CTSA. (**X**) Clinical ROC analysis in the CTSA.

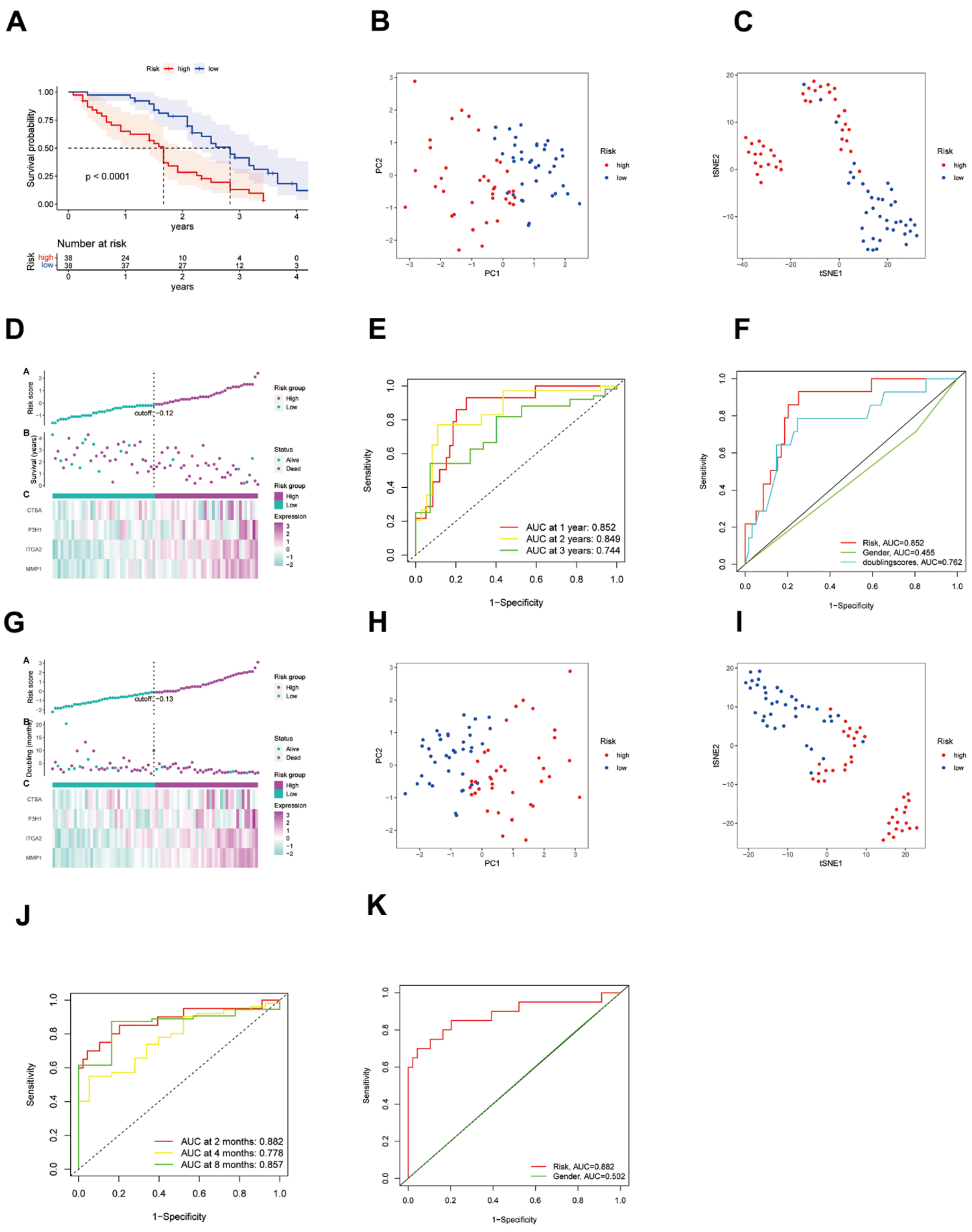


**Supplementary Figure 4. The GEO-GSE54236 dataset was used to validate the risk analysis model.** (**A**–**F**) Using the GEO-GSE54236 dataset, we demonstrated through Kaplan-Meier curve, PCA and t-SNE analysis, Risk Score Triad Diagrams, ROC curves and Clinical ROC analysis that our risk model has good predictive value for the prognosis of hepatocellular carcinoma. (**G**–**K**) We further illustrated the predictive value of the model using tumor doubling time as the endpoint event.
