# Supplementary material for: Comprehensive evaluation of genes related to basement membrane in hepatocellular carcinoma
Source: Aging (Albany NY). 2024 Jun 12;16(11):10108–31. doi: 10.18632/aging.205923 (PMC11210257; doi:10.18632/aging.205923)
Supplement: Supplementary Table 1 [file aging-16-205923-s002.pdf]

SUPPLEMENTARY TABLE

Supplementary Table 1. Cox regression analysis of all 66 common genes.

Gene	HR	z	P-value	Lower	Upper
CTSA	1.208736081	1.953452323	0.050766021	0.999368267	1.461966486
MEP1A	1.141371898	2.39743743	0.016510201	1.024422359	1.271672565
CSPG4	1.012235234	0.102779146	0.918138257	0.802723461	1.276429828
ECM1	1.14633304	2.098923067	0.035823683	1.009082487	1.302251754
NPNT	1.007033479	0.100735389	0.919760518	0.878658105	1.154164994
SMOC1	0.798636763	-2.523830863	0.011608377	0.670680112	0.951005803
SERPINF1	0.911291972	-1.658511915	0.097214185	0.81654932	1.017027433
LAMC1	1.055998131	0.793713324	0.427362342	0.923060811	1.20808081
MPZL2	1.183457869	1.813153702	0.069808118	0.986454024	1.419805174
SEMA3B	0.95708511	-0.6029272	0.546557121	0.829899768	1.103762098
LAMA4	1.062529559	0.580777097	0.561390693	0.865859198	1.303871422
LAMB2	0.914523064	-0.89420185	0.371213903	0.751863239	1.11237309
ADAMTS9	1.15147831	1.203592992	0.228746929	0.9151772	1.448792975
MATN2	0.895918432	-1.444349248	0.148640759	0.771786861	1.040014903
COL18A1	0.824274663	-2.053736731	0.040001179	0.685450377	0.991215035
ADAMTS10	0.914344947	-0.853764883	0.393235276	0.744443173	1.123022836
GPC5	0.905059229	-0.773022665	0.439508967	0.702802825	1.165522076
NTN1	0.879202179	-1.181412318	0.237438967	0.710120348	1.088542913
CTSD	1.105473087	1.028377246	0.303772407	0.913167749	1.338276288
NTN4	1.033783681	0.374077212	0.708346872	0.868612205	1.230363438
LOXL2	1.040852407	0.406449874	0.68441208	0.858096209	1.262531779
COL9A3	1.027544405	0.273398916	0.784546586	0.84567506	1.248526243
LAMB3	1.030025767	0.465960975	0.641243434	0.909505611	1.166516257
LAD1	1.036862133	0.807651529	0.419291214	0.949664335	1.132066397
COL4A5	1.076875164	0.847288172	0.396834518	0.907318578	1.278117903
LOXL4	1.061932196	1.250305779	0.211187868	0.966468678	1.166825179
VTN	0.907834236	-1.998720916	0.045638559	0.825709732	0.998126786
ITGA7	0.836451544	-1.476105543	0.139915561	0.659869621	1.060287007
SPOCK1	1.073046995	0.665152555	0.505952923	0.871758887	1.320812291
DAG1	1.017861896	0.152430931	0.878847063	0.810634681	1.278063799
ITGAV	1.209718378	2.625821436	0.008644015	1.049462999	1.394445115
SDC1	1.025940946	0.256446377	0.797606181	0.843561124	1.247751698
ADAMTS16	1.10684722	0.971784005	0.331158015	0.901920945	1.35833498
FBLN1	1.072513695	1.211194296	0.225820948	0.957645396	1.201160293
SDC4	1.022396952	0.268471839	0.788336149	0.869746271	1.20183962
ANG	0.891216004	-2.290837663	0.021972804	0.807588427	0.983503403
TINAG	1.122051271	2.110226018	0.034838892	1.008233759	1.248717417
MEGF9	1.060015695	0.542360358	0.587570283	0.858696231	1.308534069
SPON2	1.074107391	1.027796918	0.304045366	0.937218997	1.230989438
ADAM9	1.305910556	3.447748995	0.000565279	1.122068525	1.519873647
EVA1A	0.918881431	-1.289501607	0.197223758	0.808008841	1.04496763
ADAM10	1.018417794	0.135188957	0.892462478	0.781655637	1.326894802
ADAMTS17	0.951728773	-0.505998903	0.61285742	0.785751184	1.15276652
P3H1	1.511152022	3.397823996	0.000679241	1.190907368	1.917513063
ADAMTS13	1.209832757	1.87216184	0.061184217	0.991106401	1.476829631

MMP17	1.067408965	0.542758362	0.587296185	0.843383003	1.350942448
ITGA5	1.205334624	2.114436612	0.034477993	1.013737267	1.433144073
ITGB5	1.263085673	2.205451578	0.027422422	1.026338049	1.554444385
VWA1	1.054276615	0.609218937	0.54237933	0.889417543	1.249693341
CD151	1.152078406	1.842717162	0.065370317	0.991032904	1.339294234
TGFBI	0.939343121	-0.787057822	0.431248019	0.803803891	1.097737283
BCAN	1.063229498	0.491762915	0.622886956	0.832727138	1.357535879
ROBO3	1.347567377	1.728408144	0.083915076	0.96082445	1.889978795
COL2A1	1.030761316	0.543729797	0.586627425	0.924118199	1.149711034
ITGA1	0.888351324	-0.785825061	0.431969983	0.661222345	1.193498798
GPC3	0.992137493	-0.332147775	0.739777676	0.946984416	1.039443509
ITGA2	1.168161502	1.547026388	0.121856883	0.959360641	1.422407003
CTSB	1.377725522	3.074126954	0.002111196	1.123148191	1.690006386
ACHE	0.939268943	-0.781950065	0.43424392	0.802763817	1.098985938
ADAM17	1.398506601	2.582043115	0.009821731	1.084162164	1.803992777
USH2A	0.721946612	-1.907458477	0.056461243	0.51655351	1.009008556
MMP1	1.449655656	4.648097194	3.35E-06	1.23955064	1.695373673
ROBO1	1.091411473	1.475357006	0.140116588	0.97167718	1.225899947
COL9A2	1.097038218	1.039747457	0.298457246	0.921302386	1.306295165
PHF13	1.378771189	2.223279867	0.026196934	1.038773513	1.83005243
TENM1	1.030695222	0.214083474	0.830481963	0.781485423	1.359376142
